# Redescriptions of two parasitoids, *Metapelma beijingense* Yang (Hymenoptera, Eupelmidae) and *Spathius ochus* Nixon (Hymenoptera, Braconidae), parasitizing *Coraebus cavifrons* Descarpentries & Villiers (Coleoptera, Buprestidae) in China with keys to genera or species groups

**DOI:** 10.3897/zookeys.926.48688

**Published:** 2020-04-13

**Authors:** Liang Ming Cao, Cornelis van Achterberg, Yan Long Tang, Zhong Qi Yang, Xiao Yi Wang, Tian Wen Cao

**Affiliations:** 1 The Key Laboratory of Forest Protection of National Forestry and Grassland Administration, Research Institute of Forest Ecology, Environment and Protection, Chinese Academy of Forestry, Beijing 100091, China; 2 State Key Laboratory of Rice Biology, Ministry of Agriculture Key Lab of Agricultural Biology of Crop Pathogens and Insects, and Institute of Insect Sciences, Zhejiang University, Hangzhou 310058, China; 3 Laboratory of Regional Characteristic for Conservation and Utilization of Plant Resource in Chishui River Basin, College of Biology and Agriculture, Zunyi Normal University, Zunyi, 563002, China; 4 Shanxi Insect Herbarium, Institute of Plant Protection, Shanxi Academy of Agricultural Sciences, Taiyuan 030031, China

**Keywords:** *
Coraebus
*, natural enemy, synparasitism, *Symplocos stellaris*, woodborer

## Abstract

Two parasitoids, *Metapelma beijingense* Yang (Hymenoptera, Eupelmidae) and *Spathius ochus* Nixon (Hymenoptera, Braconidae) are redescribed and illustrated. Both were reared from *Coraebus cavifrons* Descarpentries & Villiers (Coleoptera, Buprestidae) boring in *Symplocos stellaris* Brand (Symplocaceae). *Metapelma beijingense* is a solitary parasitoid with a parasitism rate of about 13.5% and *S. ochus* is a gregarious parasitoid with a parasitism rate of about 21.2%. A revised key to Oriental and Palaearctic species of *Metapelma* Westwood and a key to the species of the *Spathius labdacus*-group are provided.

## Introduction

**Figure 1.**
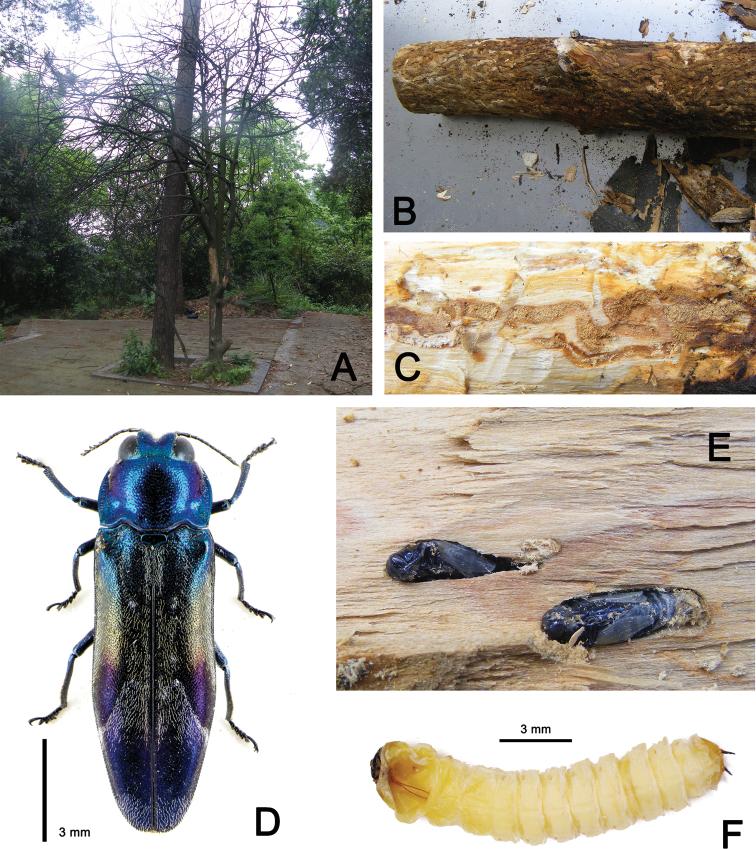
**A** Tree (*Symplocos stellaris* Brand) damaged by *Coraebus cavifrons* Descarpentries & Villiers **B** trunk damaged by *C. cavifrons* Descarpentries & Villiers **C** gallery of larvae **D** adult of *C. cavifrons* Descarpentries & Villiers **E** pupal chamber **F** larva of *C. cavifrons* Descarpentries & Villiers.

*Symplocos stellaris* Brand (Symplocaceae) is common landscape ornamental tree in South China. It is popular for its beautiful clusters of small white flowers in the spring. In addition, the wood is made into kitchen tools, furniture, etc., the seed oil is used to make soap, and the leaves and roots are used in traditional Chinese medicine. During recent investigations of woodborer biodiversity and their natural enemies in Guizhou Province, South China, we found a beautiful but little-known beetle, *Coraebus cavifrons* Descarpentries & Villiers (Coleoptera, Buprestidae) (Fig. 1D), which feeds on this tree and can cause serious damage (Fig. 1A). According to our investigation, this pest infests healthy trees rather than stressed trees. It bores into the main trunk making long longitudinal galleries (Fig. 1C). In the worst observed instance, the whole trunk was crowded with galleries bored by dozens of individual larvae (Fig. 1B). The pupal chambers are constructed about 5–10 mm under the bark in the xylem and close each other. The shape of pupal chamber is elongate-oblong. The young pupa is yellow but turns blue before emergence.

*Coraebus cavifrons* was described based on one female from Tonkin, northern Vietnam (Descarpentries and Villiers 1967), and nothing new has been reported about it except for the occurrene records by Bellamy (2008) in several provinces of southern China (Zhejiang, Fujian, Guangdong, Hainan, Sichuan). Here, we newly report this species from Zunyi City in Guizhou Province, and, more importantly, for the first time we report *Symplocos stellaris* as its host plant.

During our investigations on the biology of *C. cavifrons*, two parasitoid species belonging to different families of Hymenoptera were discovered parasitizing the buprestid larvae.

One of the parasitoid species belongs to *Metapelma* Westwood (Eupelmidae). Members of this genus are solitary parasitoids, with one larva parasitizing a single host larva (Yang 1996). Prior to this study, 38 valid species were reported (Noyes 2019), including 11 species from the Oriental region, five species from the Palaearctic region (including one extinct species from Baltic amber), 13 species from the Afrotropical region, six species from the Australian region, two species from the Nearctic region, and one species from the Neotropical region. In the Palaearctic region, Yang (1996) described two species parasitizing bark beetles in Beijing, China. The specimens found on *C. cavifrons* belong to *M. beijingense* Yang despite some minor differences with the original description by Yang (1996). The detailed redescription is given below, including the observed variation.

The second discovered parasitoid belongs to *Spathius* Nees (Braconidae), which is a huge cosmopolitan genus of the subfamily Doryctinae. The genus includes about 425 described species, of which 299 are known from the Oriental region and 91 from the Palaearctic region (Nixon 1943; Belokobylskij 2003; Chen and Shi 2004; Belokobylskij and Maeto 2009; Tang et al. 2015; Yu et al. 2016). Nixon (1943) tried to arrange the species in almost 40 species groups to facilitate identification. Our specimens belong to the *S. labdacus* species-group in the sense of Nixon (1943). Prior to this study, eight species have been included in this group, including three species occurring in China.

## Material and methods

### Survey site

Material was collected in Zunyi City, Guizhou Province, 27°41'54.91"N, 106°54'40.29"E, South China. The collection area was a small hilly, public park planted with various trees, such as *Camphora officinarum* (Lauraceae), *Osmanthus fragrans* (Oleaceae), *Magnolia liliflora* (Magnoliaceae), and *Symplocos stellaris*, which, although not the main tree, was still numerous.

### Survey methods

Dying *Symplocos* trees were cut down and cleaned of all small branches because borers are only present in the tree trunk. The trunks were cut into logs of 50 cm length. Each log was dissected and all parasitized hosts were collected and reared individually in vials (with diameter 12 mm and length 50 mm) in the laboratory at 25 °C and 65%–85% humidity. After the parasitoids emerged, they were collected, killed, and glued to triangle cards for taxonomic study. Some newly killed specimens were used for imaging. The parasitism rates were based on the number of beetles found in these dissected logs.

### Identification and photography

The parasitoid specimens were examined with a Nikon SMZ1500 stereomicroscope, and redescription of the parasitoids is based on naturally dried specimens. Photographs of fresh specimens of all the species were taken with a UV-C Optical Totally focused System (Beijing United Vision Technology Co. Ltd.) mounted on an Olympus CX31 microscope. Terminology follows Nixon (1943), van Achterberg (1993) (*Spathius*), and Gibson (2009) (*Metapelma*). Measurements were obtained using a calibrated micrometer. Specimens are deposited in Insect Museum, Chinese Academy of Forestry, Beijing, China, except for three specimens of *S. ochus* deposited in Naturalis Biodiversity Center, Leiden, the Netherlands, and two specimens of *S. ochus* deposited in Shanxi Insect Herbarium, Institute of Plant Protection, Shanxi Academy of Agricultural Sciences, Taiyuan, China. Abbreviations used in descriptions are as in Belokobylskij and Maeto (2009): POL = postocellar line, OD = ocellar diameter, OOL = ocellar-ocular line.

### Species determination and development of keys

The identification of *Metapelma beijingense* is based on the key provided by Narendran et al. (2013), the original description of *M. beijingense* (Yang 1996) and its type series. According to personal communication with Dr Gary Gibson (Agriculture and Agri-Food Canada, Canadian National Collection of Insects and Arachnids) the main morphological differences between the types and the reared specimens belong to intra-specific variation. We describe the Guizhou population to distinguish it from the holotype (Beijing population) and to facilitate future research on this species. The Guizhou population may be in the process of speciation considering the distinct differences. The key to Oriental and Palaearctic species of *Metapelma* provided is based on Narendran et al. (2013), to which *M. beijingense*, *M. zhangi* Yang, and *M. nobilis* (Förster) have been added. The inclusion of the latter species is based on the original description and additional published information only (Yang 1996).

*Spathius ochus* Nixon was identified based on Nixon’s (1943) original description, Chao’s (1957) redescription, and the keys by Belokobylskij and Maeto (2009) and Tang et al. (2015). The key to species of the *Spathius labdacus*-group provided here is based on Belokobylskij and Maeto (2009) and Tang et al. (2015), plus original descriptions and collected specimens.

**Figure 2.**
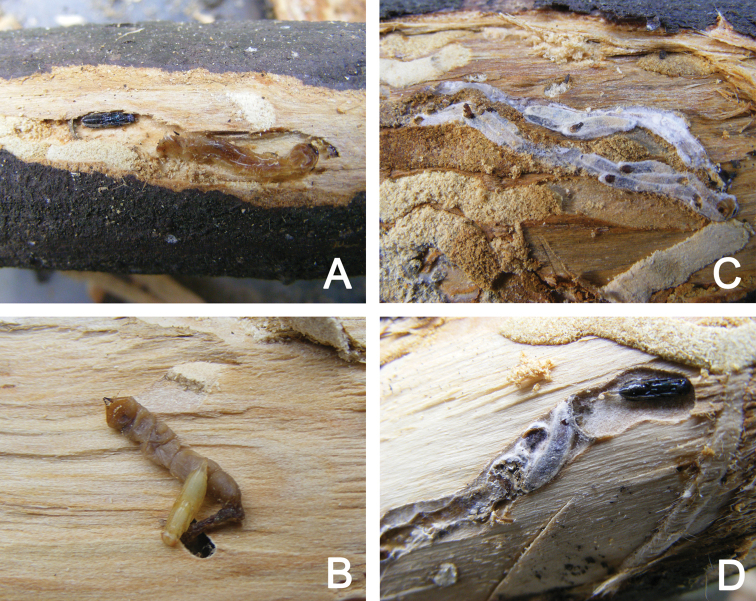
**A** A mature pupa of *Metapelma beijingense* Yang, near the buprestid larva **B** a new pupa of *M. beijingense* Yang, with the buprestid larva **C** empty cocoons of *Spathius ochus* Nixon **D** pupa of *M. beijingense* Yang and cocoons of *S. ochus* Nixon showing synparasitism.

## Taxonomy

### Metapelma

Taxon classificationAnimaliaHymenopteraEupelmidae

Westwood, 1868

4F9BEE90-F21F-534F-9321-ED901C6DBB50

#### Main history of Oriental and Palaearctic species.

Westwood (1835) established *Metapelma* with *M. spectabile* Westwood as the type species from North America. Förster (1856) subsequently described *Halidea* based on *H. nobilis* from Germany, but Ashmead (1896) synonymized *Halidea* under *Metapelma* (see Gibson 1989 for further remarks on generic synonymy).

Until now, 15 valid extant species of *Metapelma* from the Oriental and Palaearctic regions are known, namely *M. albisquamulatum* Enderlein from the Philippines, *M. beijingense* from China, *M. compressipes* Cameron from Malaysia, *M. gloriosum* Westwood from the Philippines, *M. kokkaricum* Narendranand & Abhilash from India, *M. mesandamna* Mani & Kaul from India, *M. nobilis* (Förster) from Germany, *M. obscuratum* Westwood from India, *M. pacificum* Nikolskaya from Russia, *M. periyaricum* Narendranand & Mohana from India, *M. rufimanum* Westwood from Malaysia (Sarawak), *M. strychnocola* Mani & Kaul from India, *M. taprobanae* Westwood from Sri Lanka, *M. tenuicrus* Gahan from the Philippines, and *M. zhangi* Yang from China.

#### Recognition.

**Figure 3.**
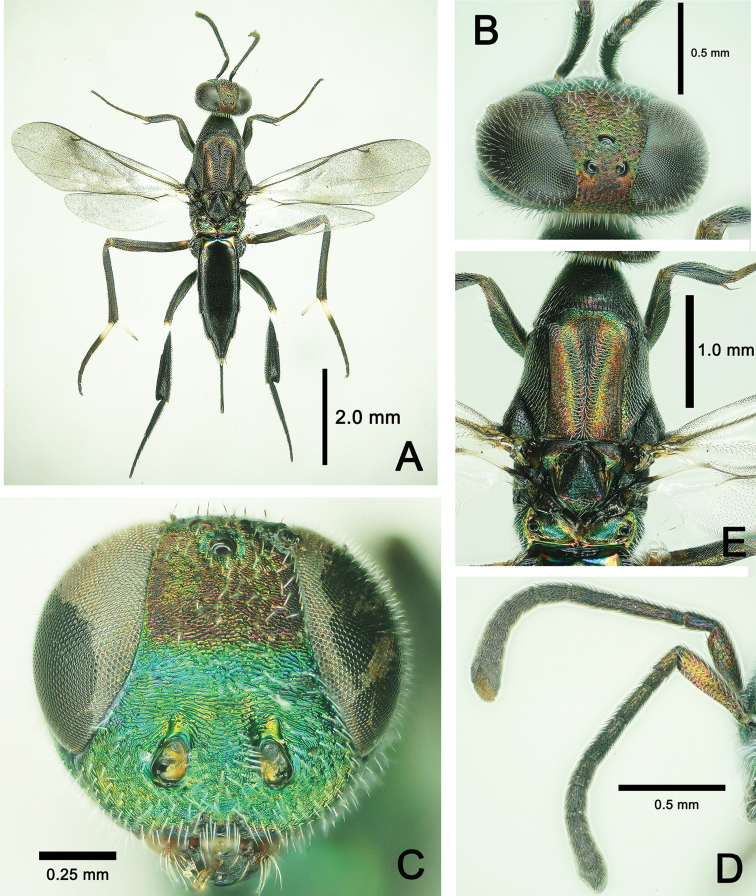
*Metapelma beijingense* Yang, ♀, China, Guizhou. **A** Habitus, dorsal aspect **B** head, dorsal aspect **C** head, anterior aspect **D** antennae, lateral aspect **E** mesosoma, dorsal aspect.

**Figure 4.**
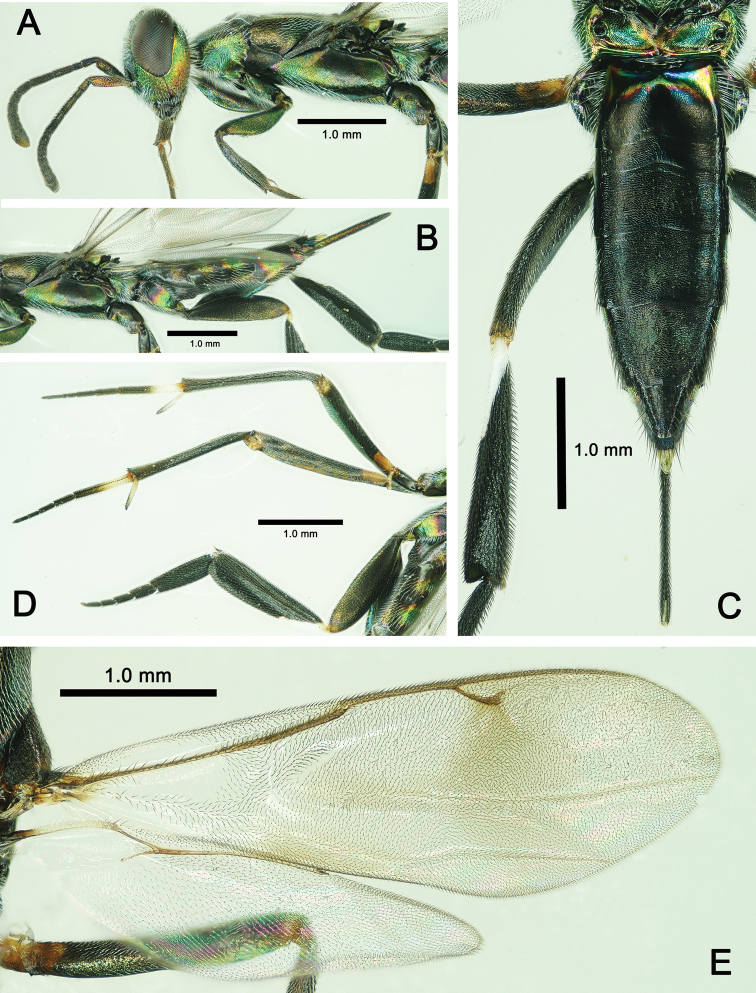
*Metapelma beijingense*Yang, ♀, China, Guizhou. **A** Head and mesosoma, lateral aspect **B** metasoma, lateral aspect, **C** metasoma, dorsal aspect **D** mid and hind legs, lateral aspect **E** wings.

*Metapelma*is one of four extant genera described for Neanastatinae (Eupelmidae). The genus is differentiated from the other three genera using the keys by Gibson (1995, 2009), but indivduals can be recognized uniquely by the following combination of characters: head lenticular with short scrobe above each torulus but scrobes not united into a common scrobal depression (Fig. 3C); antenna 13-segmented with flagellum composed of longer than wide anellus, seven funicular segments, and 3-segmented clava (Fig. 3D); scutellum entire, not divided longitudinally (Fig. 3E); mesopleuron with upper and lower mesepimeron differentiated posteriorly behind acropleuron (Fig. 4A, B); hind tibia usually conspicuously compressed and widened apically (Fig. 3A).

### Metapelma beijingense

Taxon classificationAnimaliaHymenopteraEupelmidae

Yang, 1996

AE89BE56-6896-5DBE-9294-6AF73E0B3704

Figures 3 , 4 , 5

Metapelma beijingense Yang 1996: 236.

#### Material.

Holotype, ♀, China, Beijing, Xishan Experimental Forest Farm, 7.viii.1989, Yang Zhong Qi leg., from apricot trunk, deposited in Insect Museum, Chinese Academy of Forestry, Beijing, China; 6♀♀, 1♂, China, Guizhou Province, Zunyi City, 27°41'54.91"N, 106°54'40.29"E, pupae collected 10.v.2015 from carcasses of *Coraebus cavifrons* Descarpentries & Villiers under bark of dead *Symplocos stellaris* Brand, emerged into adults 15–18.v.2015, Tang Yan Long.

#### Redescription

(based on specimens from Guizhou; differences between Beijing and Guizhou populations are shown in the key below).

**Female.** Body length 5.3–5.9 mm; forewing length 3.1–3.2 mm (Fig. 3A).

***Color*.** Body generally dark with metallic tints (Fig. 3A). In frontal view, head with lower half of frons and entire face, gena, and occiput bright metallic green, but upper half of frons and vertex with slight red tint (Fig. 3C). Propleuron, apical half of mesopleuron in lateral view with metallic green tint (Fig. 4A); V-shaped sulcus on mesonotum, apical half of axilla, metanotum bright metallic green (Fig. 3E), basal 1/3 of 1^st^ gastral tergite in dorsal view (Figure 4C), and basal 2/3 of visible ovipositor sheath in lateral view with metallic green tint (Fig. 4B). Lateral stripe on metacoxa and tergites 2–5 in lateral view varies in color from base to apex in metallic red, golden and green, successively (Fig. 4B). Apical spur and basal 1/2 of 1^st^ tarsal segment of mid leg white to pale yellow. Basal 1/5 of mesofemur yellow to brown. Outer margin of metatibia with basal 0.3–0.4 length white (Fig. 4C). Fore wing subhyaline, infuscation paler posteriorly and extend beyond medial fold toward posterior margin, as well as along medial fold and along posterior margin of discal area basally; veins and setae dark brown; hind wing subhyaline (Fig. 4E).

***Head*.** Head with sparse long white setae. In dorsal view, head width 1.75× its median length, eye occupy 1/3 of maximum width in dorsal view (Fig. 3B), pilose, nearly as long as head; ocelli small, POL: OD: OOL = 2: 3: 4. In frontal view, head as wide as high; height of eye 0.6× and mandible width 0.3× head width; minimum width of frons 1/3 head width; face, gena, frons and vertex with tiny transverse strigate-rugose stripes. Scrobes short, no more than 1/3 length of scape. In lateral view, malar sulcus straight, 1/2 eye height. Mandibles bidentate (Figs 3B, C, 4A). Dorsal margin of torulus slightly above lower ocular line, but its ventral margin distinctly below it (Fig. 3C); antenna with a very short radicle, almost not evident; scape 3.8× its maximum width, 3× length of pedicel, 4.7× length of anellus, and 2.2× length of 1^st^ funicular segment; pedicel 0.8× of length 1^st^ funicular segment; 1^st^ funicular segment 0.9× length of 2^nd^ funicular segment (Fig. 3D).

***Mesosoma*.** Mesosoma length 2.4× its maximum height. Pronotum and mesonotum with evenly distributed, dense, long white setae. Pronotum campaniform, length 1/2 of mesoscutum, with posterior margin incurved (Fig. 3E). Mesoscutum as long as broad, V-shaped notauli differentiate a convex anteromedial lobe; scutellar-axillar complex with deep oblique crenulate scutoscutellar sutures. Scutellum with an inverted Y-shaped longitudinal carina. Metanotum wide bilaterally and narrowest medially with a deep fossa, with posterior margin carinate. Medial length of propodeum twice length of medial length of metanotum and 1/2 length of scutellum, smooth without carinae, but posterior margin carinate; spiracle oval, large, situated at about half length at lateral margin of propodeum (Fig. 3E). In lateral view, mesopleuron entirely delicately reticulate, anterior lower margin with long setae, metapleuron shiny with dense setae (Fig. 4A).

***Legs*.** Profemur curved, 4.6× as long as its maximum width and 1.2× length of tibia; tarsus 1.6× length of tibia, tibia with one spur 0.4× as long as basitarsus; relative lengths of protarsal segments 1–5 = 15: 10: 7: 6: 7. Mid leg: femur 1.2× length of tibia; tarsus as long as tibia, relative lengths of segments 1–5 = 30: 9: 7: 5: 6 (Fig. 4D). Hind leg: femur 3.6× its maximum width, 0.9× length of metatibia 4.3× its maximum width, with two equally long spurs apical-ventrally, spurs 1/6 as long as basitarsus; dorsal margin of metatibia evenly curved; metatarsus as long as metatibia, relative lengths of segments 1–5 = 60: 20: 15: 12: 15. Metafemur, metatibia and metatarsal segments 1–3 compressed (Fig. 4D).

***Wings*.** Fore wing extending beyond apex of metasoma to about middle of visible part of ovipositor sheath; basal cell bare but disc with dense setae except for slender, oblique bare band behind parastigma; submarginal vein 2.3× length of marginal vein, marginal vein 0.7× length of postmarginal vein and 2.25× length of stigmal vein; R fold and Cu fold visible. Hind wing about 0.8× as long as fore wing (Fig. 4E).

***Metasoma*.** Metasoma sessile, 0.8× as long as head plus mesosoma combined; metasoma reticulate. Posterior margins of tergites 1–4 incurved medially; median length ratio of tergites 1–6 = 45: 20: 42: 49: 56: 35. Visible part of ovipositor sheath 0.5× length of metasoma, about 0.29× length of forewing, and 0.75× length of metatibia (Fig. 4B, C).

**Figure 5.**
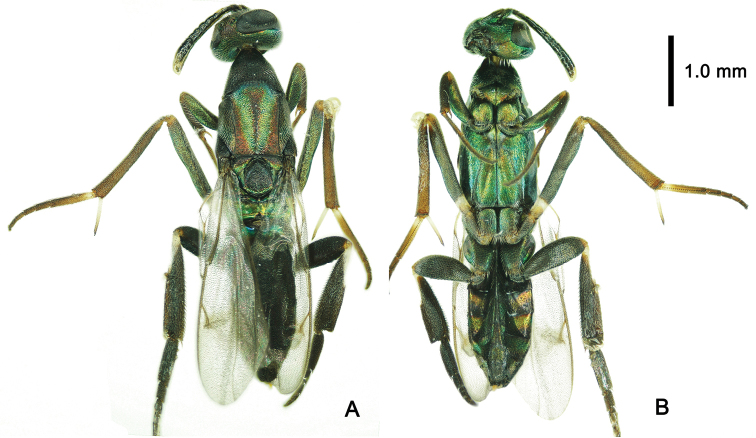
*Metapelma beijingense* Yang, ♂, China, Guizhou. **A** Habitus, dorsal aspect **B** habitus, ventral aspect.

**Male.** Body length 5.0 mm, forewing 3.2 mm (Fig. 5A, B), otherwise similar to female.

#### Remarks.

*Metapelma beijingense* is a solitary parasitoid with a parasitism rate of about 13.5%, based on seven individuals together with 34 buprestid pupae. The ratio of females to males is six.

### Key to Oriental and Palaearctic species of *Metapelma*

(Modified from Narendran et al. 2013)

**Table:** 

1	Metatibia with a dorsal forked expansion (Mani et al. 1973: fig. 38E)	***M. strychnocola* Mani & Kaul**
–	Dorsal margin of metatibia evenly curved (Fig. 4D)	**2**
2	Metatibia elongate, 5.75× wider than long	***M. compressipes* Cameron**
–	Metatibia less than 5.0× wider than long	**3**
3	Head metallic green with spot on vertex and two oval and anteriorly contiguous spots on middle of frons cupreous	***M. albisquamulatum* Enderlein**
–	Head without cupreous spots	**4**
4	Metafemur totally black or black with apex white or yellow	**10**
–	Metafemur with different color pattern	**5**
5	Fore wing apex and adjoining area infuscated	***M. gloriosum* Westwood**
–	Forewing apex subhyaline	**6**
6	Metasoma red with a cupreous tint basally; mesosoma black	***M. taprobanae* Westwood**
–	Metasoma and mesosoma partly or completely with different color pattern	**7**
7	Flagellum with 3^rd^ funicular segment as long as 2^nd^ segment; middle tibial spur as long as basal tarsal segment; metatibial lamellar width equal to hind tibial width	***M. rufimanum* Westwood**
–	Flagellum with 3^rd^ funicular segment shorter than 2^nd^ segment; mesotibial spur shorter than basal tarsal segment; width of metatibial lamella greater than width of metatibia	**8**
8	Head in anterior view with minimum distance between eyes 0.46× maximum distance between eyes; 2^nd^ funicular segment 4.5× as long as 1^st^ funicular segment	***M. periyaricum* Narendran & Mohana**
–	Head in anterior view with minimum distance between eyes less than 0.34× maximum distance between eyes; 2^nd^ funicular segment 2.7–3.1× as long as 1^st^ funicular segment	**9**
9	Malar space 0.2× height of eye; metasoma half as long as body; ovipositor sheath 0.9× as long as metasoma	***M. kokkaricum* Narendran and Abhilash**
–	Malar space 0.3× height of eye; metasoma 0.4× as long as body, ovipositor sheath as long as metasoma	***M. mesandamna* Mani & Kaul**
10	Length of ovipositor sheath equal to combined length of mesosoma and metasoma and 0.7 × length of body	***M. tenuicrus* Gahan**
–	Length of ovipositor sheath less than half of metasoma and less than 1/3 length of body	**11**
11	Spur of mesotibia black	***M. nobilis* (Förster)**
–	Spur of mesotibia white or pale	**12**
12	Metafemur black with white tip; metatibia entirely white; visible part of ovipositor sheath as long as metasoma	***M. obscuratum* Westwood**
–	Metafemur entirely black, metatibia black with white base; visible part of ovipositor sheath less than 1/2 length of metasoma	**13**
13	Head between lateral ocelli with a longitudinal carina; metatibia with dorso-basal white stripe extending 1/2 length of tibia	***M. zhangi* Yang**
–	Head between lateral ocelli without a longitudinal carina; metatibia with dorso-basal white stripe extending 2/5 length of tibia; *M. beijingense*, s.l.	**14**
14	Pedicel longer than 1^st^ funiculus (15: 14); 1^st^ funiculus 1.8× length of anellus; ovipositor sheath 0.37 × length of metasoma;1^st^ tergite 5.2× longer than 2^nd^ tergite; body length 4 mm (Yang 1996: 237, figs 373, 374)	***M. beijingense* Yang** (Beijing population; holotype)
–	Pedicel shorter than 1^st^ funiculus (12: 16); 1^st^ funiculus 3.0× length of anellus; ovipositor sheath 0.49× length of metasoma;1^st^ tergite 2.3× longer than 2^nd^ tergite; body length 5.8–6.6 mm (Figs 3A, D, 4C)	***M. beijingense* Yang** (Guizhou population)

### *Spathius* Nees, 1818

#### Spathius labdacus

Taxon classificationAnimaliaHymenopteraBraconidae

-group (sensu Nixon 1939)

78BDE61A-09B0-53B4-8907-72C55FEE774C

##### Main history

**of *S. labdacus*-group.**Nixon (1939) described the first species of this group, *S. labdacus* from Coimbatore in South India, with its host as the cotton stem weevil, *Pempheres affinis* (Faust). Nixon (1943) keyed the three described species of this group, with two new species, *S. tereus* Nixon from Philippines and *S. ochus* Nixon from Malaya and the Philippines. Chao (1957) redescribed *S. ochus* and synonymized *S. tereus* with *S. ochus*. Chao (1978) described *S. deplanatus* Chao and keyed *S. ochus* Nixon and *S. deplanatus* Chao. Belokobylskij (2003) included *S. alexandri* Belokobylskij, *S. polonicus* Niezabitowski, and *S. udaegae* Belokobylskij. Belokobylskij and Maeto (2009) reviewed and keyed Japanese species, described *S. parochus* Belokobylskij & Maeto and *S. tsukubaensis* Belokobylskij & Maeto. Tang et al. (2015) included and keyed *S. deplanatus*, *S. ochus*, and *S. parochus* as members of the *S. labdacus*-group in China.

The species group is now represented by eight valid species, namely *S. alexandri* Belokobylskij, *S. deplanatus* Chao, *S. labdacus* Nixon, *S. ochus* Nixon, *S. parochus* Belokobylskij & Maeto, *S. polonicus* Niezabitowski, *S. tsukubaensis* Belokobylskij & Maeto, and *S. udaegae* Belokobylskij.

##### Recognition.

Body slightly depressed to distinctly depressed dorso-ventrally. Eyes obliquely placed, transverse diameter usually longer than length of temple. Gena smooth. Vertex, face, and temple usually sculptured. Pronotal carina free, distinct, prominent or sharp. Setae on mesoscutum sparse and erect, posteriorly mesoscutum always with raised rugosity. Propodeum elongate, medio-longitudinal carina 0.5–1.0× anterior fork of areola. Forewing strongly infuscated, subbasal cell distinctly constricted just beyond middle and crossed by a broad subhyaline fascia at its narrowest part, base of marginal cell with an oblong subhyaline spot, a broad subhyaline fascia from base of pterostigma to posterior margin of wing. Hind coxa simple, hind femur narrowed basally. First metasomal tergite densely rugulose with short rugulae, tergite 2+3 evenly shagreened all over. Ovipositor sheath less than, equal to or longer than metasoma.

#### Spathius ochus

Taxon classificationAnimaliaHymenopteraBraconidae

Nixon, 1943

9E531062-9A35-5485-8BCA-BDE05A155682

Figures 6 , 7 , 8

Spathius ochus Nixon 1943: 372; Chao 1957: 13; Shenefelt and Marsh 1976: 1410; Chao 1978: 180; Chen and Shi 2004: 150; Yuet al. 2012; Tang et al. 2015: 79.

##### Material.

71♀♀, 5♂♂, China, Guizhou Province, Zunyi City, 27°41'54.91"N, 106°54'40.29"E, collected 10.v.2015 pupae from carcass of *Coraebus cavifrons* Descarpentries & Villiers under bark of dead *Symplocos stellaris* Brand, emerged into adults 15–20.v.2015, Tang Yan Long.

##### Redescription.

**Figure 6.**
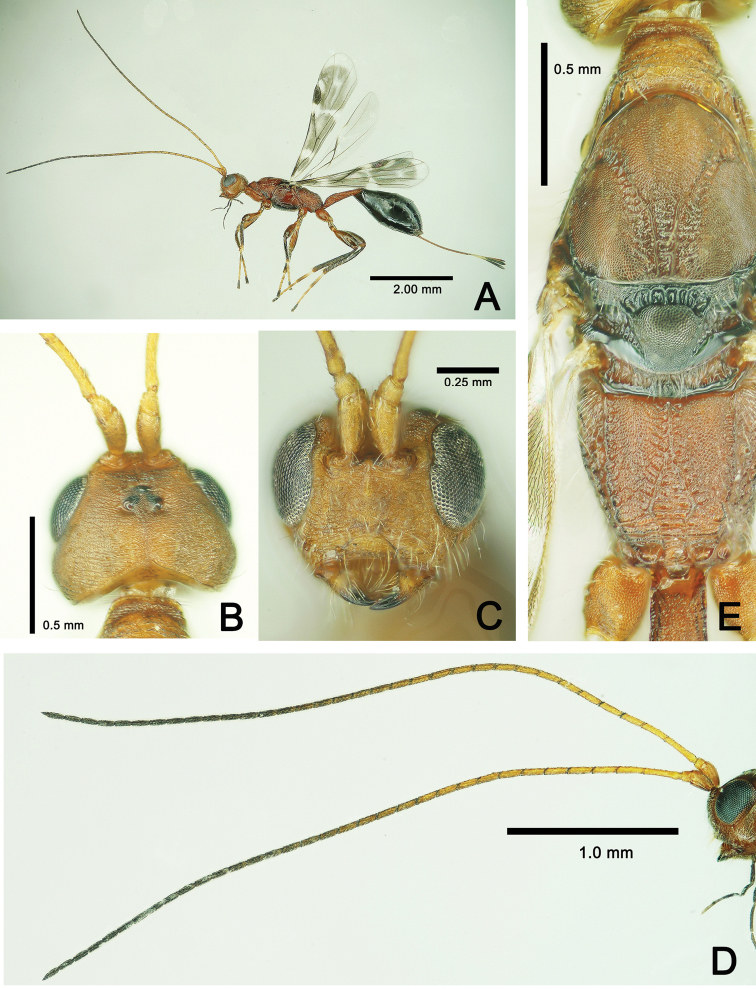
*Spathius ochus* Nixon, ♀, China, Guizhou. **A** Habitus, lateral aspect **B** head, dorsal aspect **C** head, anterior aspect **D** antennae, lateral aspect **E** mesosoma, dorsal aspect.

**Female.** Body length 4.1–4.6 mm (Fig. 6A), forewing length 3.1–3.2 mm.

***Color*.** Body generally brown (Fig. 6A). Head yellowish brown, basal half of antenna yellow, its apical half brown; pronotum, mesoscutum, propodeum, petiole and legs (except tarsi) dark brown; scutellum, axilla, metanotum, mesosternum, metasoma except first tergite, and telotarsus black; basal half of basitarsus white, and remainder of tarsus yellow. Fore wing distinctly infuscate, with several subhyaline spots and strips, apical 2/3 pterostigma dark brown, veins brown; hind wing subhyaline. Ovipositor sheath pale brown in basal 3/5, yellow in next 1/5 and dark 1/5 apically.

**Figure 7.**
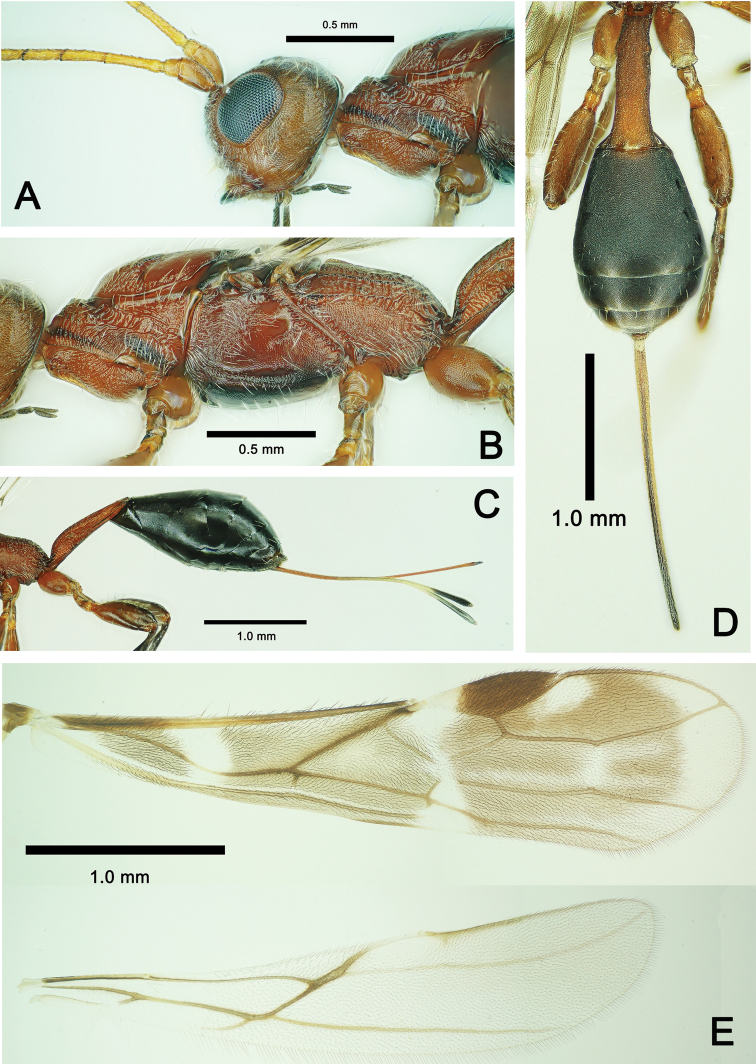
*Spathius ochus* Nixon, ♀, China, Guizhou. **A** Head, lateral aspect **B** metasoma, lateral aspect **C** metasoma, lateral aspect **D** metasoma, lateral aspect **E** wings.

***Head*.** Median length 0.8× of its width in dorsal view; with transverse striae. Length between posterior margin of lateral ocellus and occipital carina 1/2 of length of head in dorsal view; occipital carina distinct, median portion concave, reversed V-shaped; length of eye: length of temple in dorsal view = 11: 14 (Fig. 6B); eyes rather large (Fig. 7A); OOL: OD: POL = 3: 2: 1.Width of head 1.2× height in anterior view and width of face 1.1× height of eye; clypeus with transverse thin carina, face covered with sparse white setae; malar space 0.4× height of eye; height of clypeus 0.4× its width, exterior margin of clypeus slightly concave; length of maxillary palp 0.6× head width, 1.5× height of eye and 3.2× length of malar space; hypoclypeal depression deeply concave (Fig. 6C); antennae 36-segmented, scape 1/3 length of first flagellar segment, and 0.65× its maximum width; first flagellar segment 7.5× its maximum width, 1.3× as long as second segment; last antennal segment acute (Fig. 6D).

***Mesosoma*.** Length of mesosoma 2.4–4.0× its height in lateral view; pronotal keel fine, weak, with fine posterior branches, mesoscutum distinctly roundly elevated above pronotum. In dorsal view pronotum with parallel longitudinal carina bilaterally, median length of mesoscutum equal to its maximum width; mesoscutum finely granulate; notauli deep and middle of mesoscutum with two parallel longitudinal carinae, between with six transverse carinae. Anterior 1/3 of mesopleuron near pronotum and tegula with short rugae and white setae, posterior 2/3 with scaly sculpture. Scutellum apical 2/3 of scutellum finely granulate; scutellar sulcus 0.3× as long as scutellum, with 7–9 longitudinal carinae and separated small depressions. Metanotum narrow, medially concave, with 9 or 10 longitudinal carinae, propodeum weakly oblique posteriorly, 1.2× longer than its apical width, 0.5× petiole, medio-longitudinal carina bifurcates at basal 1/3 of propodeum, posterior half of propodeum with irregular carinae (Figs 6E, 7B).

***Legs*.** Fore femur 0.8× length of tibia and 3.6× its maximum width, fore tibia 6.5× longer than wide, outside with a row of spines and apex with comb of spines, ratio of fore tarsal segments I–V = 20: 10: 7: 5: 6; mid femur 0.7× length of tibia, ratio of mid tarsal segments I–V = 10: 6: 5: 4: 8; hind coxa simple, hind femur 2.5× longer than wide, 0.7× as long as hind tibia, ratio of hind tarsal segments I–V =18: 9: 6: 4: 8.

***Wings*.** Forewing 3.5× its width; pterostigma 3.5× its maximum width; 1–R1 1.25× pterostigma, r originate from middle of pterostigma; SR1 7.2× longer than r, straightly extending to wing margin; r nearly 1/4 of 2–SR, cu-a perpendicular to CU1, m–cu enters second submarginal cell; meeting point of 2–SR, 2–M and 2–SR+M weak, veins reduced; 1–SR+M straight, 1–SR 1/4 length of 1–M; M+CU1 distinctly curved, apical subbasal cell narrow and elongate, r–m unsclerotized, hardly invisible; 3–M and CU1a reaching wing margin. Length of hind wing 4.5× its width, m-cu and SR pigmented (Fig. 7E).

***Metasoma*.** First tergite 3.5–3.9× longer than its maximum apical width in dorsal view, with regular longitudinal carinae; in lateral view first tergite slender and 1.5–1.7× as long as propodeum, spiracular tubercles located at basal third, laterally with erect white long setae; tergites 2–4 densely granulate; fifth and sixth tergites smooth. Length of visible setose part of ovipositor sheath 0.7–0.8× length of metasoma, 0.85× length of fore wing, and 0.6× length of body (Fig. 7C, D).

**Figure 8.**
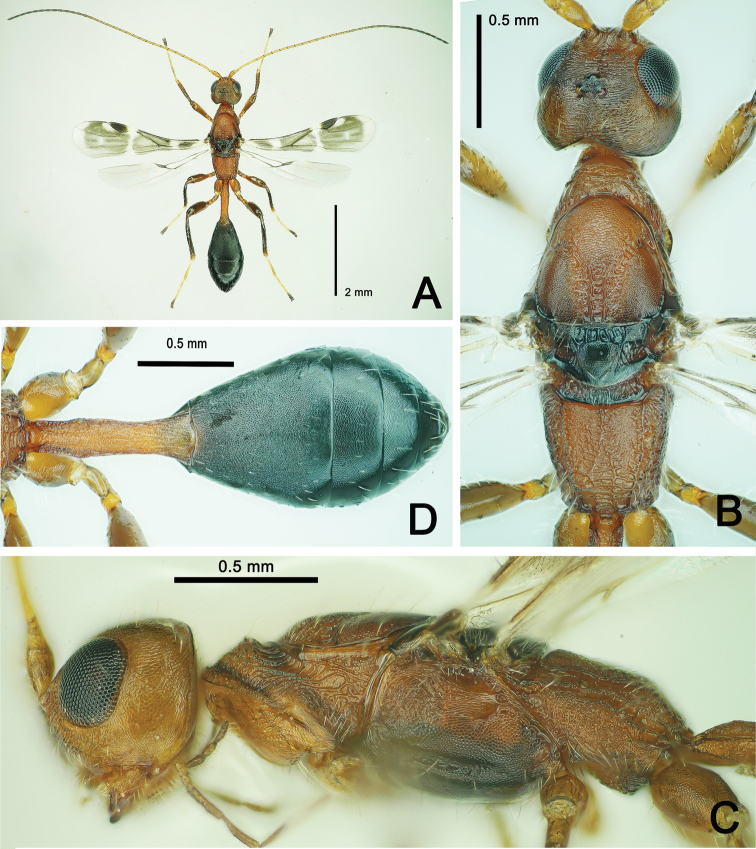
*Spathius ochus* Nixon, ♂, China, Guizhou. **A** Habitus, dorsal aspect **B** head and mesosoma, dorsal aspect **C** head and mesosoma, lateral aspect **D** metasoma, dorsal aspect.

**Male.** Body length 4.0–4.2 mm, forewing 2.7 mm (Fig. 8), otherwise similar to female.

##### Remarks.

The mesosoma is variably depressed; usually 2.4–2.9× longer than high, but in some specimens up to 3.7–4.0×. Obviously, this character is useless to separate *S. tereus* Nixon, 1943. Therefore, we agree with Chao (1957) that the latter cannot be separated. *Spathius ochus* is a gregarious koinobiont ectoparasitoid like most other *Spathius*, each buprestid larva can feed 3–9 individuals. From one tree 11 borer larvae were parasitized by 42 individuals of *S. ochus*, together with seven borer larvae were parasitized by *M. beijingense* and 34 live buprestid pupae, resulting in a parasitism rate of about 21.2% for *S. ochus*. The sex ratio is about 14:1 (71 females to 5 males).

The very interesting phenomenon of synparasitism (Tobias 2007) is shown in Figure 2D; one individual of *M. beijingense* and four individuals of *S. ochus* were together parasitizing the same woodborer larva. Likely, these two ectoparasitoid species laid their eggs near the host at about the same time, and the larvae did not start fighting each other because the host was large enough to avoid severe food competition. Of course, this is only circumstantial evidence that is in need of corroboration.

The species is very similar to *S. parochus* Belokobylskij & Maeto and can be recognized with the key below.

#### Key to species of *Spathius labdacus*-group

**Table:** 

1	Mesosoma very strongly depressed, 4.0–6.0× longer than its maximum height and ovipositor sheath 0.3–0.4× as long as metasoma	***S. deplanatus* Chao**
–	Mesosoma less depressed, 2.0–4.0× longer than its maximum height, if 3.5–4.0× (= *S. tereus* Nixon,1943) then ovipositor sheath 0.6–0.8× as long as metasoma	**2**
2	Ovipositor sheath 0.5–0.8× as long as metasoma	**3**
–	Ovipositor sheath as long as metasoma or longer	**5**
3	Ovipositor sheath 0.4–0.5× as long as metasoma; base of hind tibia pale; medio-longitudinal carina of propodeum 1.4–1.8× as long as anterior fork of areola	***S. tsukubaensis* Belokobylskij & Maeto**
–	Ovipositor sheath0.7–0.8× as long as metasoma; base of hind tibia dark brown; medio-longitudinal carina of propodeum 0.5–1.0× as long as anterior fork of areola	**4**
4	First metasomal tergite 1.5× as long as propodeum (Fig. 7A), medio-longitudinal carina of propodeum about as long as anterior fork of areola (Fig. 6E)	***S. ochus* Nixon**
–	First tergite 1.6–1.8× as long as propodeum, medio-longitudinal carina of propodeum 0.5–0.7× length of anterior fork of areola	***S. udaegae* Belokobylskij**
5	Ovipositor sheath about 1.8× as long as metasoma	***S. alexandri* Belokobylskij**
–	Ovipositor sheath less than 1.2× as long as metasoma	**6**
6	Pronotal keel sharp and protuberant	***S. labdacus* Nixon**
–	Pronotal keel fine and hardly protruding	**7**
7	Length of first metasomal tergite about twice its maximum width	***S. polonicus* Niezabitowski**
–	Length of first tergite3.0–3.3× its maximum width	***S. parochus* Belokobylskij & Maeto**

## Discussion

During the investigation, we found that the host *C. cavifrons* boring in *Symplocos stellaris* has only one generation per year in Zunyi, Guizhou Province. From the end of May to early June, this buprestid begins emerging and it will last for about 2 weeks. We chose 20–30 days before its emergence to cut and dissect logs when there are no emergence holes of parasitoids in the trunk. The best time for collecting these parasitoids proved to be the first week of May. We guess that both two parasitoids are at least oligophagous, because there are no *C. cavifrons* larvae available for laying eggs after their emergence. However, they may search for another host to lay their eggs. Both parasitoids seem to have two generations per year in Zunyi, but this needs to be further investigated.

Combined study of the stressed tree (host), the woodborers (pest), the parasitoids (natural enemies) and their relationships is interesting, and biological traits may be useful in their taxonomy. For the identification of *Metapelma beijingense* we used only morphological and biological evidence, but a molecular data analysis study with fresh material of the Beijing and Guizhou populations might be helpful for the identification and determination of the systematic status of these two populations.

## Supplementary Material

XML Treatment for Metapelma

XML Treatment for Metapelma beijingense

XML Treatment for Spathius labdacus

XML Treatment for Spathius ochus
